# Silver Diamine Fluoride for Arresting Severe Early Childhood Caries: A Review of the Clinical Framework for Interim Stabilization, Exit Criteria, and Timely Definitive Care

**DOI:** 10.3390/children13040490

**Published:** 2026-03-31

**Authors:** Ziad D. Baghdadi

**Affiliations:** 1 Centre for Community Oral Health, College of Dentistry, University of Manitoba, Winnipeg, MB R3B 0L8, Canada; drziadeddin.albaghdadi@gmail.com or zalbaghdadi@atsu.edu; 2 Department of Preventive Dental Sciences Division of Pediatric Dentistry, College of Dentistry, University of Manitoba, Winnipeg, MB R3E 0W2, Canada; 3 TopSmiles Pediatric Dentistry and Orthodontics, Winnipeg, MB R2M3A4, Canada; 4 Butterfly Dental Group, Winnipeg, MB R3Y1P5, Canada

**Keywords:** severe early childhood caries, silver diamine fluoride, interim stabilization, definitive restorative care, care pathways

## Abstract

**Highlights:**

**What are the main findings?**

- Silver diamine fluoride is most appropriately used as a short term interim stabilization strategy for arresting severe early childhood caries within a defined care pathway, not as an open-ended substitute for durable restorative care.
- Clinical guidelines and systematic reviews support the use of silver diamine fluoride to arrest cavitated lesions in primary teeth. However, the current evidence does not establish it as a universal long-term solution for severe disease.

**What is the implication of the main finding?**

- Clinicians should document treatment intent, triage teeth by prognosis, monitor actively, and define exit criteria for transition to definitive restorative care when durability, function, symptoms, or follow-up reliability warrant it.
- Systems should judge the value of buying time by whether children move toward timely, definitive care and child-centered outcomes, rather than normalizing prolonged temporization due to access delays.

**Abstract:**

Early childhood caries (SECC) in children aged 3–4 years is a high-burden condition with consequences that extend beyond the dentition, including pain, infection, sleep disturbance, impaired nutrition, disrupted family functioning, and diminished quality of life. In contemporary pediatric practice, 38% silver diamine fluoride (SDF) and other minimally invasive approaches are widely used to stabilize disease while behavior matures, preventive strategies are intensified, or access to definitive care is secured. This perspective argues that SDF should be conceptualized not as a standalone solution, but as an evidence-supported interim stabilization strategy embedded within a defined, goal-directed care pathway. Its use is most appropriate when framed as a means of buying time under clear clinical intent, particularly in cases where teeth are expected to remain functional for years, symptoms are present, structural integrity is compromised, or follow up is uncertain. Although clinical guidelines and systematic reviews support SDF for arresting cavitated lesions in primary teeth, current evidence does not support its use as a universal long-term treatment for severe disease. Real-world data suggest that many SDF-treated teeth require additional intervention within approximately 2 years, and that delays to definitive care—including treatment under sedation or general anesthesia when indicated—are often relatively short. In response, this paper proposes a practical bridge-to-destination framework grounded in three principles: explicit treatment intent, child-centered outcomes, and predefined exit criteria to ensure a timely transition to definitive dental care. Rather than discouraging SDF use, this approach seeks to optimize its role within a continuum of dental care, emphasizing proportionality, transparency, and durable outcomes for children.

## 1. Why Is This Perspective Needed

Severe early childhood caries (SECC) in 3- to 4-year-olds is not simply a technical challenge, it is a preventable, progressive condition with broad clinical and social consequences, including pain, infection, disrupted sleep, impaired nutrition, developmental effects, and strain on family functioning and quality of life [1]. In this context, 38% silver diamine fluoride (SDF) has become an important tool in pediatric dentistry. Its ability to arrest cavitated lesions in primary teeth and slow short-term disease progression makes it especially valuable when immediate definitive care is not feasible [2,3,4,5,6,7,8,9].

The concern, however, is not the use of SDF itself, but how its role is sometimes interpreted. A subtle shift has emerged in which short-term disease control is treated as an endpoint rather than a phase of care. In some settings, interim stabilization risks are being viewed as sufficient in themselves, without adequate consideration of tooth prognosis, structural compromise, symptom burden, follow-up reliability, or the child’s need for durable function over time [2,10,11,12]. Yet minimal intervention is a means of delivering care, not the measure of success. For children with SECC, meaningful outcomes include sustained freedom from pain and infection, restoration of function, and acceptable psychosocial impact within the realities of family capacity to engage in ongoing care [1,11].

This perspective is directed toward pediatric dentists, dental public health clinicians, trainees, and policymakers working in high-income systems where delays in definitive treatment can occur. It advances a clear position: in SECC, SDF should be understood as a time-limited, purpose-driven intervention within a structured care pathway. It serves as a bridge toward definitive care, not a substitute for it, when long-term tooth retention, functional demands, symptoms, or follow-up uncertainty require more durable solutions.

For clarity, this approach is described as a preservation to precision pathway: protect the child from immediate harm while deliberately progressing toward the level of care needed for lasting outcomes. The aim is not to challenge the value of minimally invasive dentistry, but to align its application with current evidence and clinical responsibility by defining its role, limits, and appropriate endpoints in the management of severe disease [2,3,4,5,10,11].

## 2. What the Evidence Supports

The evidence base for silver diamine fluoride (SDF) is robust in several key respects and provides clear guidance for clinical use. Authoritative recommendations, including those from the American Dental Association and the American Academy of Pediatric Dentistry, support SDF and other nonrestorative therapies for arresting cavitated lesions in primary teeth when delivered within an ongoing caries management plan and an established dental home [2,3,4,5,13]. Systematic reviews and meta-analyses consistently demonstrate that SDF can arrest dentine caries in primary teeth, particularly with repeated applications, although study protocols, outcome measures, and follow-up durations vary [6,7,8,9].

This body of evidence supports confident use of SDF for its well-established role: arresting active cavitated lesions, reducing immediate biological risk, and stabilizing disease in the short term. It is especially appropriate when time is needed to optimize prevention, support behavioral adaptation, coordinate referral, or sequence care in a manner proportionate to the child’s needs. Importantly, this evidence also underscores the need for precision in clinical communication. When SDF is used, it should be clearly described as an intervention aimed at lesion arrest and interim stabilization, rather than definitive resolution [2,3,4,5,6,7,8,9,10,11,12,13,14,15,16,17,18,19,20,21,22,23,24,25,26,27,28].

At the same time, restorative care remains firmly supported by evidence and clinical guidelines. Recommendations from the American Dental Association and contemporary staged caries management frameworks endorse conservative operative treatment for moderate-to-advanced cavitated lesions when durability is required to achieve sustained health outcomes [10,11]. Minimal intervention and restorative care should not be viewed as competing philosophies. In many cases, a well-executed conservative restoration represents the most reliable and least harmful option for restoring function, controlling symptoms, and preserving the tooth over time (Table 1).

## 3. SDF Treatment Limitations

The most important limitation is conceptual: lesion arrest is not equivalent to durable function. SDF does not rebuild lost tooth structure, restore form, or ensure that a structurally compromised tooth will remain functional and symptom-free until exfoliation [10,29]. In the context of SECC, where primary teeth in a 3–4-year-old must function for several years, this distinction is substantive rather than semantic. It marks the difference between a biologically useful bridge and an overextended treatment plan.

Observational studies provide valuable insights but should be interpreted with appropriate caution. A matched-cohort study from private practice found that children treated with SDF had different short-term care patterns, including more visits and fewer restorations within 1 year [14]. A private claims analysis reported that approximately 40% of SDF-treated primary teeth required additional treatment within about 24 months [12]. A retrospective cohort study identified delays in access to sedation or general anesthesia among children who ultimately received those services, although the delays were measured in weeks to months rather than years [15]. A naturalistic study conducted in community clinics demonstrated meaningful but variable tooth survival, with outcomes differing by age and tooth type [16]. Collectively, these findings support SDF as a useful stage of care, but they do not justify claims of universal long-term sufficiency.

Evidence on caregiver decision-making and implementation further reinforces this interpretation. Acceptance of SDF is generally favorable but remains variable, influenced by factors such as staining, tooth location, quality of explanation, and family priorities [17,20]. Clinician adoption depends on training, workflow integration, and organizational support [18,19]. These findings underscore a practical point: SDF is most effective when its interim role is clearly communicated and when follow-up care is feasible.

Accordingly, the most defensible approach in SECC is to use precise, disciplined language. It is appropriate to state that SDF can arrest lesions and stabilize selected cases. But greater caution is warranted when describing long-term function, multi-year tooth survival without additional intervention, or the avoidance of definitive restorative care in high-burden disease [2,3,4,5,6,7,8,9,10,11,12,14].

## 4. SDF Practical Treatment for Interim Stabilization

In SECC, the central question is not whether SDF is effective, but under what circumstances SDF alone can protect a child’s dental health for a clinically appropriate duration. A practical framework should make that judgment explicit and guide decision-making accordingly (Table 2).

Practice Pathway at a Glance

Stabilize the disease immediately

→ Triage each tooth individually
→ Define the intended treatment endpoint
→ Establish monitoring intervals and clear exit criteria
→ Transition to definitive care when indicated.

Key Steps in Applying the Framework

1. State the immediate treatment objective. Clearly explain why SDF is being used as a temporary measure, such as arresting lesions, reducing short-term risk, supporting behavior acclimatization, reinforcing prevention, or bridging to referral and definitive care.
2. Triage teeth by prognosis, not ideology. SECC is a diagnosis, not a complete treatment plan. Management should be individualized at the tooth level. Some teeth are appropriate for ongoing non-restorative care, others may require interim restorations, and some warrant timely definitive treatment where durability is the primary goal [10,11].
3. Define the intended SDF outcome. Specify the planned course after SDF application, whether continued monitoring, definitive chairside restoration, or treatment under sedation or general anesthesia. Lack of clarity at this stage increases the risk of protocol drift.
4. Establish exit criteria in advance. Define clear thresholds for transitioning from SDF to definitive care. These should include the onset of pain or infection, failure of lesion arrest, recurrent structural breakdown, unacceptable esthetic or psychosocial impact, unreliable follow-up, or a time point at which durability becomes necessary [10,11,12,17,20].
5. Use the time gained by using SDF to prepare for permanent dental care. SDF should create a window to move treatment forward, not simply defer it. Any period of stabilization should be used to strengthen prevention, complete referrals, improve child cooperation, or secure definitive care. The value of time depends on measurable improvement in the child’s oral health trajectory.

## 5. SDF Ethics, Access, and Equity

The central ethical question in SECC is not whether interim stabilization can be appropriate; it often is. The concern arises when a temporary measure becomes the de facto endpoint for children who lack timely, definitive care. Clinically meaningful outcomes remain clear: freedom from pain and infection, preservation of function over time, and an acceptable psychosocial impact within the family’s realistic capacity for follow-up [1,11]. A rights-based pediatric perspective reinforces this standard by placing the child’s best interests and access to appropriate treatment at the forefront, rather than treating them as secondary considerations [23,24].

This issue is particularly salient in high-income settings, where access barriers are well-documented. American Academy of Pediatric Dentistry policy statements and survey data describe limited access to operating room time in hospitals and delays in pediatric dental treatment [21,22]. Broader literature on oral health and social determinants similarly demonstrates how systemic constraints disproportionately affect children and families with the least capacity to navigate delays [25,26]. In this context, SDF may be both necessary and compassionate in the short term, but it should not be framed in a way that recasts structural limitations as clinical success.

Clinical judgment must also account for the care setting. Some children with SECC cannot safely or humanely receive definitive dental treatment in a conventional chairside environment, even with appropriate behavior guidance. In such cases, referral for sedation or general anesthesia may be the appropriate next step [15,27]. Interim stabilization is justified precisely because it protects the child during this transition, not because it replaces the need for definitive care.

Similar challenges arise in other constrained high-income systems, where limited access can normalize extraction, containment, or prolonged temporization as substitutes for comprehensive treatment [28]. Accordingly, this framework is simultaneously clinical, ethical, and policy-relevant, emphasizing that the appropriate use of SDF must remain aligned with both patient-centered care and equitable access to definitive treatment.

## 6. What This Perspective Asks of Clinicians and Systems

A perspective piece should conclude with practical advice, rather than rhetoric. In applied terms, this manuscript encourages clinicians and systems to adopt the following approach:

- Use precise dental definitions. Describe dental outcomes accurately, referring to lesion arrest and interim stabilization with SDF when the evidence from dental exams, dental training, and clinical experience supports it [2,3,4,5,6,7,8,9].
- Differentiate temporary SDF treatment from permanent dental restorative care. Every interim dental treatment plan should include a clearly stated purpose, defined review intervals, and a permanent restorative treatment plan [10,11,12].
- Prioritize child-centered health and welfare outcomes. The focus should remain on whether the temporary use of SDF treatment can reliably avoid pain, preserve tooth function, save teeth, and improve quality of life [1,11].
- Acknowledge SDF limitations and treatment access constraints. When dental restorative care is delayed, this should be acknowledged directly and incorporated into the treatment plan, rather than treated as an unstated assumption [21,22,25,26].
- Prioritize SDF outcomes that will improve care. Research and service evaluation should focus on clinically meaningful endpoints such as pain, infection, sleep, eating, quality of life, revisit burden, wait times, and equity of access, rather than relying solely on billing data or survival proxies [1,12,14,15,16,21,22,29].

## 7. Summary

SDF has a clear and valuable role in contemporary pediatric dentistry for arresting SECC. The dental literature and professional guidelines support its use as an effective interim measure to arrest caries within a comprehensive treatment plan. However, there is no reliable evidence to support SDF as a long-term substitute for durable restorative care when function, symptom control, structural integrity, or reliable follow-up are at risk. The central issue is not whether SDF is effective, but how far its role should be extended before definitive treatment is provided. Interim use must not become a default endpoint. When temporary treatment is allowed to replace necessary restorative care, it risks compromising both oral health and overall child well-being. Accordingly, SDF should be used with clear clinical objectives, defined limits, and an explicit plan for transition to definitive restorative treatment. In children at high risk for SECC, SDF is best understood as a means to arrest disease and stabilize conditions until appropriate restorative care can be delivered. Prolonged reliance on SDF as a substitute for definitive treatment may introduce avoidable dental and welfare risks [1,2,10,11,12,21,22,25,26,27,28,30]. Definitive treatment follows the principles of minimal intervention dentistry, adopting a pathway that prioritizes preservation before precision [31,32,33].

## 8. Conclusions

SDF is a valuable tool for interim stabilization in SECC, but it is not a substitute for definitive restorative care. Its success depends on its use as a purposeful bridge within a defined care pathway, guided by clear intent, exit criteria, and a commitment to timely, durable, and child-centered outcomes. Prolonged temporization should not become a default endpoint.

## Figures and Tables

**Table 1:** What can be stated with confidence about SDF in SECC, and what should not be inferred from the current evidence base.

Evidence Domain	Supported Conclusion	Meaning for This Perspective
Guidelines and systematic reviews	Clinical guidelines and systematic reviews support the use of SDF to arrest cavitated lesions in primary teeth as part of a comprehensive caries management plan.	SDF is well-suited for interim stabilization of caries; however, it is not established as a universal long-term substitute for durable restorative care, particularly in children with SECC.
Restorative guidance	Evidence-based conservative dental care supports the use of SDF for managing cavitated lesions when sufficient durability can achieve child-centered health outcomes.	Restorative treatment should align with the principles of minimally invasive dentistry. The central consideration is which approach best preserves the child’s overall well-being over the expected lifespan of the affected tooth, along a preservation-to-precision pathway.
Real-world pathway studies	Use of SDF may alter treatment pathways, often increasing the need for ongoing monitoring and, in some cohorts, leading to additional treatment within approximately two years. When sedation or general anesthesia is required, access is frequently delayed by weeks to months.	In practice, dental care pathways prioritize the prevention of pain, control of infection, and timely provision of definitive treatment, including the use of sedation or general anesthesia when indicated.
Acceptability and implementation studies	Parental and caregiver acceptance of SDF for arresting caries is generally favorable, particularly when the clinical rationale is clearly explained, and treatment is limited to posterior teeth. Successful implementation depends on effective communication, appropriate clinician training, and familiarity with reimbursement structures.	SDF use is associated with tooth staining, which may raise esthetic concerns. Its application also requires ongoing dental supervision to ensure that caries is effectively arrested and that disease progression or infection does not occur.
Access and equity literature	Barriers to SDF care include delays in treatment and the high, often prolonged, cost of dental procedures. Often, equity is used as an excuse for failing to deliver proper, timely care.	While SDF can be effective in arresting caries, it should not be used as a substitute for definitive treatment, including the removal of decayed tissue and appropriate restoration of the tooth.

**Table 2:** Clinical decision-making considerations for selecting SDF treatment or dental restorative treatment.

Decision Factor	SDF Interim Stabilization Is Reasonable When	Restorative Dental Care Should Be Prioritized When
Symptom status	No pain or signs of infection, with a reasonable expectation that comfort can be maintained between dental appointments.	Pain, infection, persistent sensitivity, or recurrent tooth breakdown indicate that comfort cannot be reliably maintained with SDF treatment.
Structural loss and function	Caries lesions are arrestable, and existing tooth structure supports short-term function while restorative dental care is being scheduled.	Structural tooth loss compromises function, hygiene, or maintainability, requiring durable restoration rather than further temporization.
Expected service time of the tooth	The tooth has limited remaining service time, or the interim period is short and purpose-specific.	The tooth lifespan is needed to function for several years, making durability and restoration of form more critical.
Follow-up reliability	Caregivers can reliably return for monitoring, prevention, and escalation of care when needed.	Recall follow-up is uncertain or inconsistent, making a monitoring-dependent approach less reliable and potentially unsafe.
Behavioral or care-setting context	Immediate dental restorative treatment is not feasible, but a staged plan is underway, including behavior guidance, referrals, and sedation planning.	An alternative setting is needed to provide the restorative treatment, and interim care is being used without a clear or credible timeline for definitive treatment.
Caregiver priorities and esthetics	Caregivers understand the temporary role of SDF and accept potential discoloration of the tooth.	Esthetic concerns, psychosocial impact, or caregiver preferences make extended interim management unacceptable or counterproductive.
Access to definitive care	Interim care is being used deliberately to prevent deterioration while dental restoration treatment is arranged.	Delays in access to dental care have become open-ended, effectively converting a temporary SDF treatment into a substitute for the dental restoration of the tooth.

## Data Availability

No new data were created or analyzed in this study. All cited data are available in the referenced public sources.
